# Methylenetetrahydrofolate Reductase C677T and A1298C Mutations in Women with Recurrent Spontaneous Abortions in the Northwest of Iran

**DOI:** 10.5402/2012/945486

**Published:** 2012-11-14

**Authors:** Ahmad Poursadegh Zonouzi, Nader Chaparzadeh, Mehrdad Asghari Estiar, Mahzad Mehrzad Sadaghiani, Laya Farzadi, Alieh Ghasemzadeh, Masoud Sakhinia, Ebrahim Sakhinia

**Affiliations:** ^1^Department of Cellular and Molecular Biology, Faculty of Sciences, Azarbaijan University of Tarbiat Moallem, Tabriz, Iran; ^2^Students' Scientific Research Center & Department of Medical Genetics, School of Medicine, Tehran University of Medical Sciences, Tehran, Iran; ^3^Department of Obstetrics and Gynecology, Women's Reproductive Health Research Center, Tabriz University of Medical Sciences, Tabriz, Iran; ^4^School of Medicine, University of Liverpool, Merseyside, Liverpool L69 3GE, UK; ^5^Tuberculosis and Lung Disease Research Center and Department of Medical Genetics, Faculty of Medicine, Tabriz University of Medical Sciences, Tabriz 51656 38464, Iran; ^6^Tabriz Genetic Analysis Center (TGAC), Tabriz University of Medical Sciences, Tabriz 51656 38464, Iran

## Abstract

*Introduction*. Recurrent spontaneous abortion (RSA) is a significant obstetrical complication that may occur during pregnancy. Various studies in recent years have indicated that two common mutations (C677T and A1298C) of the methylenetetrahydrofolate reductase (MTHFR) gene are risk factor for RSA. This study was carried out to determine the influence of (C677T and A1298C) of the methylenetetrahydrofolate reductase (MTHFR) gene mutations with RSA. *Materials and Methods*. A total of 139 women were included in this study: 89 women with two or more consecutive miscarriages and 50 healthy controls. Total genomic DNA was isolated from blood leukocytes. To determine the frequency of the two common C677T and A1298C MTHFR gene mutations in the patients and controls, we used two methods, amplification refractory mutation system-PCR and PCR-restriction fragment length polymorphism. *Results*. There is no significant difference in the prevalence of 677T/T genotype among women with RSA and healthy controls (*P* = 0.285). Also no statistically significant difference in the frequency of A1298C MTHFR gene mutation was detected between the two groups (*P* = 0.175 ). *Conclusion*. In conclusion, the results indicate that the Amplification Refractory Mutation System-PCR method was in complete concordance with the results obtained by standard PCR-restriction fragment length polymorphism method. The results also show no significant difference in MTHFR C677T/A1298C genotype distribution among the two groups; therefore, further studies on larger population and other genetic variants to better understand the pathobiology of RSA are needed.

## 1. Introduction

Recurrent spontaneous abortion (RSA) represents a significant clinical problem, which an estimated 1% to 5% of all women of reproductive age experience [1, 2]. However, the pathogenesis of RSA is complicated, and the cause in 40%–50% of the cases is not well understood [2]. Genetic, anatomic, endocrine, immunologic, infectious, and environmental factors have been proposed as causes of RSA [3, 4]. Various reports have postulated thrombophilia as a risk for RSA [5, 6]. Deficiency in the homocysteine metabolism pathway resulting in an elevation of homocyteine level in plasma (hyperhomocysteinemia) has been regarded as a cause of Thrombophilia [5]. Methylenetetrahydrofolate reductase (MTHFR) is one of the main regulatory enzymes in the metabolism of homocysteine that catalyses the reduction of 5,10-methylenetetrahydrofolate to 5-methyltetrahydrofolate [7]. Mutations in MTHFR gene lead to decreased activity of enzyme and hyperhomocysteinemia, which induces platelet aggregation through promotion of endothelial oxidative damage [8]. Although several mutations within the MTHFR gene were described, C677T and A1298C mutations are the two most common mutations [9]. C677T transition is a missense mutation in the exon 4 of this gene, which converts an alanine to a valine codon (at codon 222) in the N-terminal catalytic domain of the protein leading to a thermolabile protein, with decreased enzymatic activity [10]. The second mutation is MTHFR A1298C, that is, associated with decreased activity of enzyme, but not with thermolability. A1298C transversion is a point mutation in exon 7, characterized by a glutamate to alanine substitution (at codon 429) within the C-terminal regulatory domain of the protein [11]. Numerous investigations have been performed on the incidence of MTHFR C677T and A1298C mutations in women with RSA [9, 12–17]. Some of these studies have demonstrated a relationship between these mutations and RSA [9, 10, 12, 13, 18], whereas others have been unable to confirm these results [14, 19]. Thus, the role of MTHFR C677T and A1298C mutations in RSA is still controversial.

Here, we evaluated the prevalence of MTHFR C677T and A1298C mutations in RSA patients in the northwest of Iran and compared it with healthy controls using two methods, PCR-restriction fragment length polymorphism (PCR-RFLP) and Amplification Refractory Mutation System-PCR (ARMS-PCR).

## 2. Materials and Methods

### 2.1. Patients

We enrolled 89 patients with a history of first trimester RSA from many different regions of Northwest of Iran that were all referred by obstetricians. The patients with chromosomal abnormality, uterine anomalies, genital infections, and endocrinological disorder were excluded from the study. Fifty healthy women with at least two live births from the same region were selected as a control group. Informed consent was obtained from all participants before enrolment. The mean age of the case and control groups were 30.17 and 31.54 years, respectively. The average abortion in the cases was 2.94 (ranged from 2 to 7), and the average successful pregnancy in the controls was 2.2 (ranged from 2 to 4).

### 2.2. Extraction of Genomic DNA

Peripheral blood samples (5 *μ*L) were obtained from all cases and controls in tubes containing EDTA. Total genomic DNA was extracted from leukocytes by a standard salting-out protocol as previously published [20]. The DNA concentration was firstly measured by spectrophotometer and adjusted to 100 ng/*μ*L by adding double distillation H_2_O and then stored in −20°C.

### 2.3. Genotype Screening

We applied two methods, PCR-RFLP and ARMS-PCR, for identification of MTHFR C677T and A1298C gene mutations.

#### 2.3.1. PCR-RFLP-Based Screening

To perform PCR-RFLP, each DNA sample was amplified by polymerase chain reaction using primers that are shown in Table 1 [21]. PCR was carried out on 25 *μ*L volume, in an eppendorf thermal cycler (eppendorf, Germany). Initial denaturation at 94°C for 5 min was followed by 35 cycles of denaturation at 95°C for 40 s; annealing at 58°C (MTHFR C677T) and 62°C (MTHFR A1298C) and extension at 72°C for 40 s and final extension at 72°C for 7 min were performed. PCR amplifications were then confirmed by electrophoresis on 2% agarose gel, and after staining with ethidium bromide, viewed with ultraviolet light. Finally, the amplified products were digested by restriction enzymes that are shown in Table 1 [21] and the digested fragments were separated by electrophoresis on 8% polyacrylamide gels that stained with silver nitrate.

#### 2.3.2. ARMS-PCR-Based Screening

ARMS-PCR was performed using three primers for each mutation, one forward primer and two reverse primers specific for the wild type and mutant alleles that are shown in Table 2 [12, 22]. PCR was carried out on 20 *μ*L volume, in an eppendorf thermal cycler (eppendorf, Germany). First denaturation step (96°C, 2 min) was followed by 10 cycles of denaturation (96°C, 15 s) and annealing/extension (65°C, 60 s), and a final 20 cycles of denaturation (96°C, 10 s), annealing (61°C, 50 s), and extension (72°C, 30 s) [22]. The PCR products were electrophoresed on 2% agarose gel and stained with ethidium bromaid.

### 2.4. Statistical Analysis

Statistical analysis was performed using SPSS version 16. The genotype distributions of each mutation, the frequency of heterozygous and homozygous were compared between patients and controls with Pearson's Chi-square test. A *P* value of <0.05 was regarded as significant. The homozygote and heterozygote genotypes of each group were unified as a new group and then odds ratios and 95% confidence intervals were calculated.

## 3. Results

### 3.1. Comparison of PCR-RFLP and ARMS-PCR Methods

Analysing MTHFR gene mutations among the RSA and control samples by using PCR-RFLP and ARMS-PCR methods clarified that there was no difference between these two methods.

### 3.2. Genotype Distribution of C677T and A1298C MTHFR Gene Mutations in Case and Control Groups

**Figure 1:**
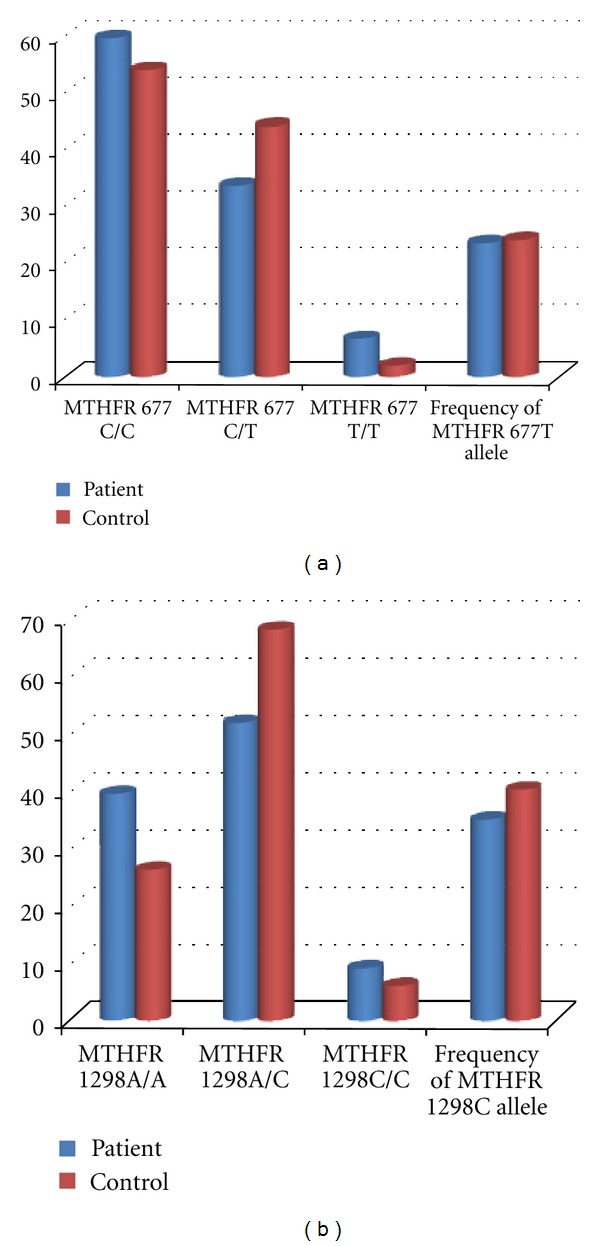
Distribution of the MTHFR gene mutations genotypes (a) MTHFR C677T and (b) MTHFR A1298C. In each graph, columns show wild type, heterozygote, homozygote, and total mutant allele frequencies, respectively.

The genotype distribution of each MTHFR mutations in patients and controls are shown in (Figure 1).

**Figure 2:**
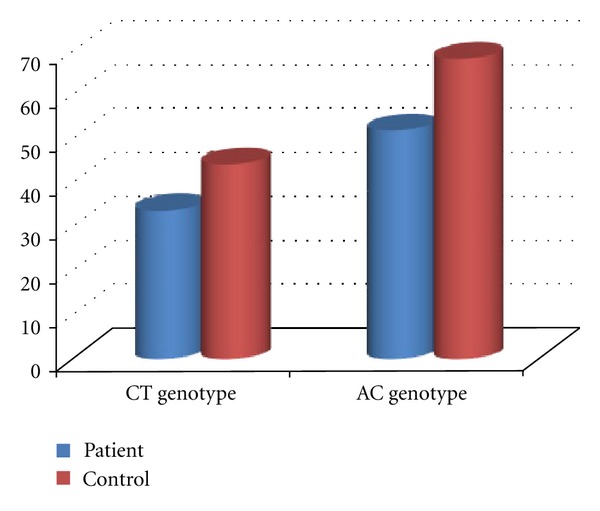
Frequency of heterozygous genotypes for MTHFR C677T and MTHFR A1298C among women experiencing RSA compared with fertile control women.

**Figure 3:**
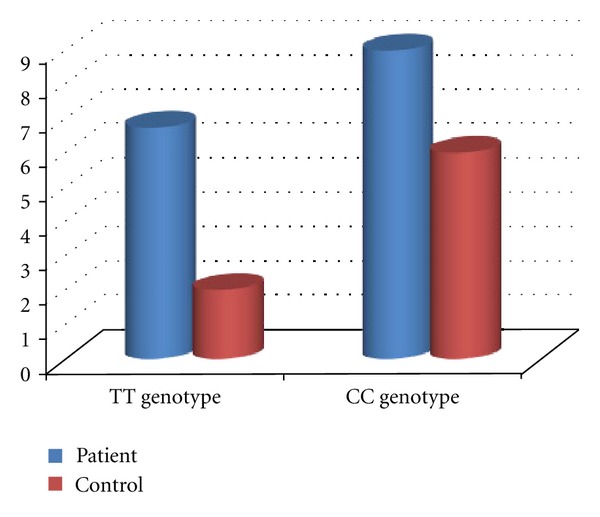
Frequency of homozygous genotypes for MTHFR C677T and MTHFR A1298C among women experiencing RSA compared with fertile control women.

The frequency of 677C/T genotype MTHFR gene was 33.70% in patients and 44% in controls while the frequency of 677T/T genotype was 6.74% in patients and 2% in controls (Figure 2). The frequency of 677T allele was 23.59% in patients and 24% in controls (Figure 3).

**Figure 4:**
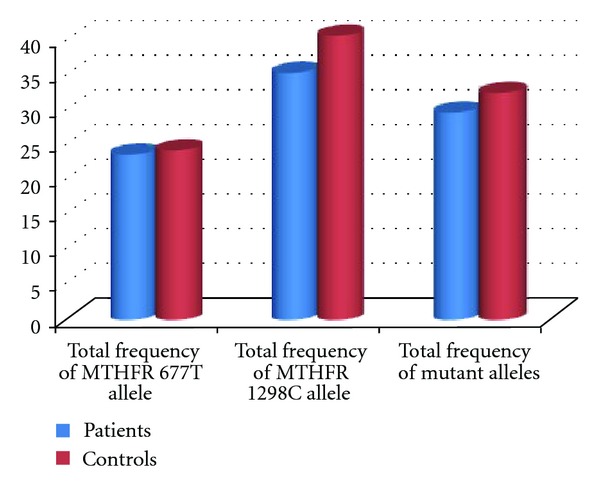
Frequency of mutant alleles for MTHFR, (677T and 1298C) alleles, and total mutant allele among women experiencing RSA compared with fertile control women.

There is no significant difference in the prevalence of 677T/T genotype among women with RSA and healthy controls (*P* = 0.285). The frequency of 1298A/C genotype MTHFR gene was 51.68% in patients and 68% in controls. The frequency of homozygote genotype was 8.98% in patients and 6% in controls. In contrast with the heterozygote genotype, the frequency of homozygote genotype was higher in RSA patients compared with the control group. The frequencies of 1298C allele were 34.83% in patients and 40% in controls. No statistically significant difference in the frequency of A1298C MTHFR gene mutation was detected between the two groups (*P* = 0.17). The frequencies of MTHFR 677T and MTHFR 1298C alleles were (23.4%, 34.8%) in patients and (24%, 40%) in controls, respectively (Figure 4). The total mutant allele frequencies were 29.21% in women experiencing RSA and 32% in fertile controls. All possible MTHFR C677T/A1298C genotype combinations were represented in both groups (Table 3).

The frequencies of 677CT/1298AC combined heterozygosity in patients were 15.73% and 32% in the control group. Our findings indicated that combined MTHFR C677T/A1298C genotype distribution has no statistically significant differences. The odds ratios (ORs) of the MTHFR 677C/T (OR = 0.69; 95% confidence interval (CI) = 0.33–1.42) and the MTHFR 1298A/C (OR = 0.50; 95% CI = 0.23–1.09).

## 4. Discussion

Although most researchers generally use the PCR-RFLP method for detection of gene mutations, here we have focused on studying the concordance between the PCR-RFLP and ARMS-PCR methods. We applied the two methods of PCR-RFLP and ARMS-PCR for detection of genotypes. The results from PCR-RFLP method were the same as ARMS-PCR method. Therefore, due to the fact that it is relatively easy to perform low costs, and the possibility of detecting a larger number of samples, we proposed ARMS-PCR as a useful method that could be used as an alternative for identification of these mutations.

Many pieces of literature have discussed the matter that MTHFR gene mutations might be a risk factor for recurrent spontaneous abortions [9, 10, 12, 13, 18]; hence, we investigated the prevalence of C677T and A1298C, two common MTHFR gene mutations in Northwestern Iran, to determine whether these mutations related with RSA. The genotypes distribution of C677T MTHFR gene mutation was compared in the two studied groups. It is clear from Table 3 and Figure 2 that the heterozygosity in nucleotide 677th of the MTHFR gene has no statistically significant difference among the two groups. However, homozygosity for 677T allele of the MTHFR gene in women with RSA was higher than healthy controls, which were concordant with previous reports [9, 10, 18]. The total frequency of 677T alleles for MTHFR gene 677T was also compared between women experiencing RSA with fertile control women (23.59%, 24%), respectively. On the whole, our data has indicated no statistically significant difference in the prevalence of C677T mutation between the two groups. These observations are in contrast with a previous report on the literature [14, 19]. This difference may be explained by differences in the populations or by using low numbers of samples. On the other hand, our findings suggest that C677T mutation probably has no significant role in the etiology of first-trimester RSA in patients in the Northwest of Iran or that other hyper coagulant gene mutations may have a role in RSA. Furthermore, the frequency of A1298C MTHFR gene mutation was also compared in patients and healthy women. As shown in Table 3 and Figure 2, the frequency of 1298A/C genotype MTHFR gene in patients was less than the control group. The frequency of 1298C/C genotype was higher in patients with RSA compared with the control group, in contrast with the heterozygote genotype. The total frequency of 1298T alleles for MTHFR gene 677T were also compared between women experiencing RSA compared with the fertile women in the control group (34.83%, 40%), respectively (Figure 2). This was in accordance with earlier investigations [13, 15], which reported no association between MTHFR 1298A/C and RSA. Findings of previous studies have shown that the presence of combined C677T/A1298C genotypes highly increased the risk of RSA [16]. Therefore, prevalence comparison of 677CT/1298AC compound heterozygosity in patients and healthy controls showed no significant difference.

## 5. Conclusion

In conclusion, our results showed no significant variations in MTHFR C677T and A1298C genotype distribution among patients who suffered from RSA and controls. Further studies on larger population may be needed. To better understand the pathobiology of RSA disease, we need to identify novel genetic variants and the interactive effects of these variants with each other and the environment. Due to the fact that, it is possibile to detect a large number of samples and low costs, we proposed ARMS-PCR as a useful method that could be used for identification of these mutations.

## Figures and Tables

**Table 1:** Sequence of primers, PCR product size, restriction enzymes, and size of digested fragments that are used for screening by PCR-RFLP.

Mutation	Sequence of primers	PCR product	Restriction	Wild type	Heterozygote	Mutant
		(bp)	enzyme			
MTHFR	F-TGAAGGAGAAGGTGTCTGCGGGA	198	Hinf1	198	198/175/23	175/23
C677T	R-AGGACGGTGCGGTGAGAGTG					

MTHFR	F-CTTTGGGGAGCTGAAGGACTACTAC	163	MboII	56/31/30/28/18	84/56/31/30/28/18	84/31/30/18
A1298C	R-CACTTTGTGACCATTCCGGTTTG					

**Table 2:** Primer pairs used for screening of MTHFR mutations by ARMS-PCR.

Mutation	Forward primer	Reverse primer	PCR
			product (bp)
MTHFR	5-TGC TGT TGG AAG GTG CAA GAT-3	RW 5-GCG TGA TGA TGA AAT CGG-3	226
C677T		RM 5-GCG TGA TGA TGA AAT CGA-3	226

MTHFR	5 -CCTTTGGGGAGCTGAAGGACTACTAC-3	RW 5-CAAAGGACTTCAAAGACAGTC-3	120
A1298C		RM 5-GGTAAAGAACAAAGACTTCAAAGACACTGTG-3	127

**Table 3:** MTHFR C677T/A1298C genotype combinations.

Genotypes	Cases (*n* = 89)	Controls (*n* = 50)
of MTHFR		
C677T/A1298C		
CC/AA	13 (14.60%)	7 (14%)
CC/AC	32 (35.95%)	18 (36%)
CC/CC	8 (8.98%)	2 (4%)
CT/AA	16 (17.97%)	5 (10%)
CT/AC	14 (15.73%)	16 (32%)
CT/CC	0 (0%)	1 (2%)
TT/AA	6 (6.7%)	1 (2%)
TT/AC	0 (0%)	0 (0%)
TT/CC	0 (0%)	0 (0%)
